# Effects of Predator Species and Size on Prey Escape Success Through the Digestive Tract

**DOI:** 10.1002/ece3.72477

**Published:** 2025-12-01

**Authors:** Shinji Sugiura

**Affiliations:** ^1^ Graduate School of Agricultural Science Kobe University Kobe Japan

**Keywords:** American bullfrog, aquatic beetle, frog, Hydrophilidae, native species, non‐native predator, post‐capture escape

## Abstract

Many animals flee from their predators before capture. However, some species can successfully escape even after being swallowed. The likelihood of postcapture escape may be influenced by predator species and size, although this hypothesis has rarely been tested. In this study, I systematically investigated the escape success of the aquatic beetle *Regimbartia attenuata* (Coleoptera: Hydrophilidae) after being swallowed by six predator species—five native frog species (Anura: Ranidae, Dicroglossidae, Hylidae) and one non‐native bullfrog, *Aquarana catesbeiana* (Ranidae)—which differed in body size, taxonomic affiliation, and their evolutionary history of coexistence with the beetle. Among adult *R. attenuata* individuals that were ingested, 79% survived passage through frog digestive tracts and successfully escaped from the vent (cloaca). Escape rates ranged from 57% to 91% across frog species. However, escape success was not significantly influenced by beetle size, frog species, or frog size. All surviving beetles escaped within 7 h after ingestion, whereas beetles that died inside the frogs were excreted much later, between 19 and 161 h postingestion. The time required for live beetles to escape through frog digestive tracts was significantly different from the time required for dead beetles to be excreted. Therefore, the active movement of adult *R. attenuata* through the frog digestive tract towards the frog's vent likely facilitated their successful escape through the cloacal aperture across various frog species and body sizes—including the non‐native *A. catesbeiana*—with consistently high frequency.

## Graphical abstract

Adults of the aquatic beetle *Regimbartia attenuata* can survive passage through the digestive tracts of six predator (frog) species, including a non‐native bullfrog. Their active movement through the gut likely facilitates their escape through the cloaca, irrespective of predator species or size.

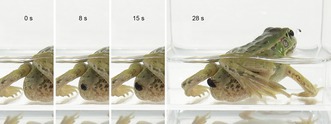

## 1 Introduction

Predation has driven the evolution of diverse defensive strategies in animals (Edmunds 1974; Ruxton et al. 2018; Sugiura 2020a). For example, some animals have evolved toxins or venoms that can deter predators (Eisner et al. 2005). In response, predators have developed counter‐adaptations; some species have evolved a high tolerance to prey toxins and venoms (van Thiel et al. 2022). Over evolutionary time, prey defences and predator counter‐defences have reciprocally influenced each other (Prudic 2009). However, recent human activities have intentionally or unintentionally introduced many animal species to new regions (Seebens et al. 2017), and the establishment of non‐native species has often disrupted native prey–predator interactions (Sugiura 2016; David et al. 2017). Some native animals are vulnerable to non‐native predators due to the lack of a shared evolutionary history (Fritts and Rodda 1998; Strauss et al. 2006; Carthey and Banks 2014), whereas others can withstand predation pressures from non‐native species by relying on pre‐existing defences (Carthey and Banks 2014) or by acquiring novel ones (Strauss et al. 2006).

Many animals flee from predators before capture (Edmunds 1974). However, some animals can successfully escape even after being swallowed by predators (Fair 1969; Norton 1988; Brown 2007; Sugiura 2020b). For example, adults of the weevil genus *Sphenophorus* Schönherr (Insecta: Coleoptera: Curculionidae) can pass through predator (toad) digestive systems and are eventually excreted alive a few days after ingestion (Fair 1969; Barrentine 1988, 1991). Similarly, several species of snails and bivalves can survive passage through predator (fish) digestive tracts and are excreted alive (Norton 1988; Brown 2007). These escapes are considered passive behaviours because prey are expelled only when the predator defecates, without actively moving through the digestive tract.

In contrast to such prey, the aquatic beetle *Regimbartia attenuata* (Fabricius) (Coleoptera: Hydrophilidae) can escape more rapidly after ingestion by predators (i.e., frogs; Sugiura 2020b). When swallowed by the pond frog *Pelophylax nigromaculatus* (Hallowell) (Anura: Ranidae), adult *R. attenuata* use their legs to move through the digestive tract and eventually escape through the frog's vent (cloaca) a mean of 90 min after ingestion (Sugiura 2020b). Because adult beetles with experimentally immobilised legs were unable to escape, Sugiura (2020b) speculated that adult *R. attenuata* may use their legs to induce defecation by the frog, facilitating their own expulsion. Although the ability to digest prey varies among predator species (Fair 1969), the effects of predator species on the escape success of *R. attenuata* have not been fully analysed (Sugiura 2020b).

Body size can strongly influence both defensive and counter‐defensive behaviours in animals (Caro 2005; Whitman and Vincent 2008; Brodie and Brodie 1990). Larger body size in prey is often associated with greater quantities of defensive chemicals (Sugiura and Sato 2018), longer protective hairs (Sugiura and Yamazaki 2014), and enhanced overall defensive capabilities (Whitman and Vincent 2008; Sugiura 2018, 2020a; Sugiura and Tsujii 2022; Sugiura and Urano 2025). Conversely, larger body size in predators may confer greater resistance to defensive chemicals, stings, or other painful defences, as well as stronger offensive traits (Brodie and Brodie 1990; Sugiura and Sato 2018; Sugiura and Tsujii 2022; Sugiura 2025a).

For example, in the bombardier beetle *Pheropsophus occipitalis jessoensis* Morawitz (Coleoptera: Carabidae), larger adults produce more defensive chemicals and more frequently survive predation by toads of the genus *Bufo* Garsault (Anura: Bufonidae) because their hot defensive discharge induces the toads to vomit them alive (Sugiura and Sato 2018). Larger toads, however, tend to show greater resistance to these chemicals (Sugiura and Sato 2018). When prey escape through the predator's digestive tract and exit via the cloaca, larger prey may exhibit greater tolerance to digestive fluids, but they may also face lower survival rates during gut passage and cloacal escape because their larger size hinders movement through narrow digestive pathways. In contrast, larger predators may facilitate prey escape by having a wider digestive tract, yet their stronger digestive enzymes may increase prey mortality, reducing escape success. Despite these plausible effects, the influence of body size on successful escape through the digestive tract has not been systematically investigated in animals exhibiting this behaviour. In *R. attenuata* , which can survive passage through frog digestive tracts, the escape success rate varied from 66.7% to 100.0% depending on frog species (Sugiura 2020b), suggesting that predator species or size can influence the outcome. However, the previous study lacked sufficient sample sizes to rigorously examine these effects, leaving the question unresolved.

**FIGURE 1:**
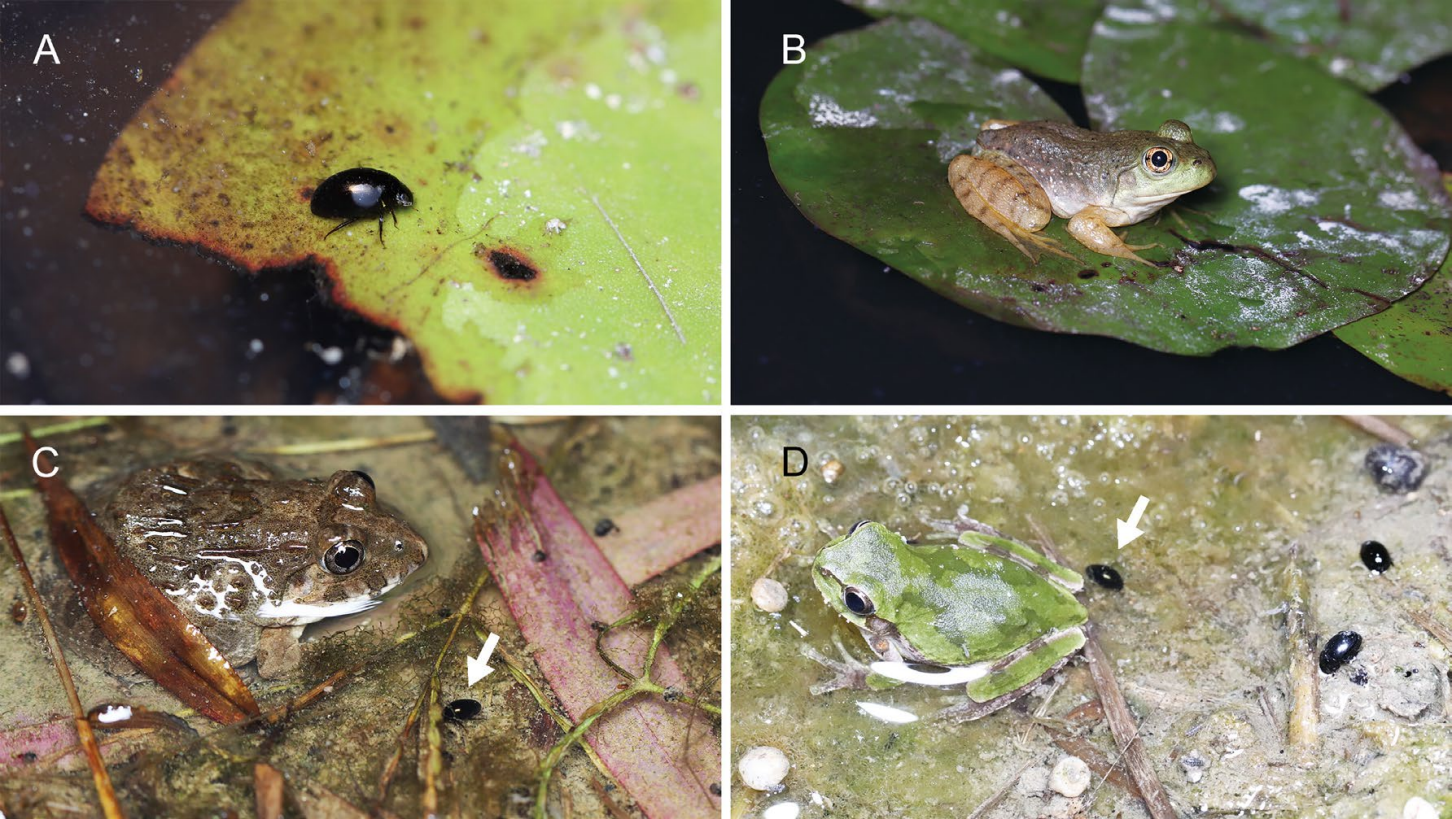
The aquatic beetle *Regimbartia attenuata* and its potential predators under field conditions. (A) An adult *R. attenuata* on a floating leaf of the aquatic plant *Nymphaea*. (B) A juvenile of the non‐native bullfrog *Aquarana catesbeiana* on a floating leaf of *Nymphaea*. (C) A native marsh frog, *Fejervarya kawamurai* , with an adult *R. attenuata* (indicated by an arrow). (D) A native tree frog, *Dryophytes japonicus* , with an adult *R. attenuata* (indicated by an arrow). Photos (A) and (B) were taken in the same pond on the same date. Photo credits: Shinji Sugiura.

In August 2024, I frequently observed adult *R. attenuata* (Figure 1A) both underwater and on the floating leaves of the aquatic plant *Nymphaea* L. (Nymphaeales: Nymphaeaceae) in a pond in central Japan, where juveniles of the American bullfrog *Aquarana catesbeiana* (Shaw) (= *Lithobates catesbeianus* ) (Anura: Ranidae; Figure 1B) were abundant. *Aquarana catesbeiana* is widely recognised as an invasive non‐native predator across the globe (Ficetola et al. 2007). As in other countries (Wu et al. 2005; Silva et al. 2009; Barrasso et al. 2009; Oda et al. 2019), *A*. *catesbeiana* has exerted impacts on native animals in Japan (Hirai 2005; Hirai and Inatani 2008; Nakamura and Tominaga 2021; Sugiura and Hayashi 2024, 2025a).

**FIGURE 2:**
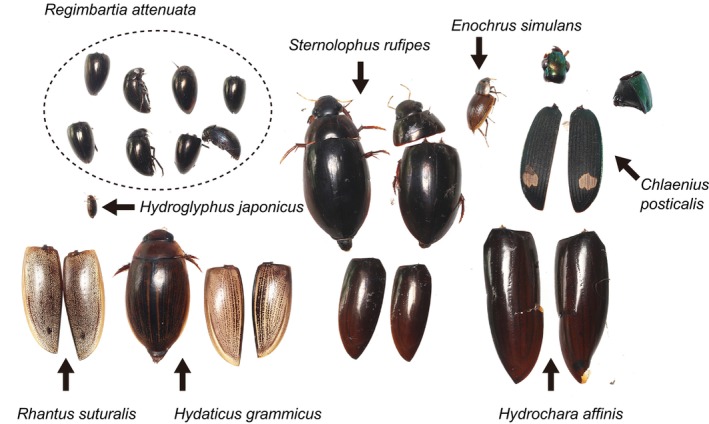
Undigested beetle remains excreted by juvenile *Aquarana catesbeiana* collected from a pond. Dead adults of *Regimbartia attenuata* are surrounded by a dotted circle. Other beetles include *Hydaticus grammicus* , *Hydroglyphus japonicus* , *Rhantus suturalis* , *Enochrus simulans*, *Sternolophus rufipes*, *Hydrochara affinis*, and *Chlaenius posticalis*. Photo credit: Shinji Sugiura.

To investigate whether *A*. *catesbeiana* preys on adult *R. attenuata* and other native insects (Sugiura and Hayashi 2024, 2025a), I collected 21 juvenile individuals (snout–vent length: 41.6–55.0 mm) from the pond between 03:00 and 04:00 on 17 August 2024. After transferring them to the laboratory, I found that they had excreted dead adult beetles, including *Hydaticus grammicus* (Germar), *Hydroglyphus japonicus* (Sharp), *Rhantus suturalis* (MacLeay) (Coleoptera: Dytiscidae), *R. attenuata* , *Enochrus simulans* (Sharp), *Sternolophus rufipes* (Fabricius), *Hydrochara affinis* (Sharp) (Coleoptera: Hydrophilidae), and *Chlaenius posticalis* Motschulsky (Coleoptera: Carabidae), by 04:30 on the same day (Figure 2). Notably, one adult *R. attenuata* was alive among the excreted beetles, indicating that it had successfully escaped from a juvenile bullfrog. This is the first evidence of *R. attenuata* escaping from a wild‐caught frog. Although successful escape from native frog species has previously been observed under laboratory conditions (Sugiura 2020b), the effects of non‐native predators on the escape success of *R. attenuata* have remained unexplored.

To examine how predator species and body size influence post‐capture prey escape, I quantitatively investigated the escape success of *R. attenuata* under laboratory conditions using six frog species (one non‐native species, *A*. *catesbeiana*, and five native species) that belonged to different families, differed in body size, and varied in their evolutionary history of coexistence with the beetle. I compared the success rate and the time required for *R. attenuata* to escape through the digestive tract between the non‐native *A*. *catesbeiana* and the native frog species. Furthermore, I investigated the effects of *R. attenuata* body size and frog size on escape success and escape time. These observations allowed me to test whether larger beetles escape more successfully and quickly due to their greater mobility and tolerance to the frog's digestive fluids, and whether larger frogs suppress beetle escape through stronger digestive forces and longer digestive tracts. Finally, I discuss the factors influencing the successful escape of *R. attenuata* from within frogs.

## 2 Materials and Methods

### 2.1 Study Species

I investigated the escape behaviour of adult *R. attenuata* from the non‐native bullfrog *A*. *catesbeiana* and five native frog species.

The hydrophilid beetle *R. attenuata* is widely distributed across Asia and Australasia (Mai et al. 2022). In Japan, adults are commonly found in shallow waters of ponds and rice paddies (Mitamura et al. 2017; Nakajima et al. 2020). They feed on live and dead leaves of aquatic plants underwater and frequently rest on vegetation and ground above the water surface (Figure 1A,C,D). I collected adult *R. attenuata* from five sites in the Kinki region of central Japan between September 2019 and October 2024. Beetles were housed in plastic containers (diameter: 80 mm; height: 80 mm) filled with 120 mL of water at 25°C (Sugiura 2020b). Leaves of *Monochoria vaginalis* (Burm.f.) C.Presl ex Kunth (Commelinales: Pontederiaceae) and filamentous green algae (*Spirogyra* Link, Zygnematales: Zygnemataceae) were provided as food. Prior to behavioural observations, body length and body weight were measured to the nearest 0.01 mm and 0.1 mg, respectively, using digital callipers and an electronic balance (PA64JP, Ohaus, Tokyo, Japan). Each beetle was used only once for the following observations.

The American bullfrog, *A*. *catesbeiana*, originally from eastern North America, has been deliberately introduced to many regions in South America, Europe, and East to Southeast Asia, as well as parts of western North America (Ficetola et al. 2007). In Japan, *A*. *catesbeiana* was imported from the United States in 1918 for human consumption (Ota 2002; Matsui and Maeda 2018). After escaping from breeding facilities, the species established populations in lakes, ponds, and rice paddies throughout the country (Ota 2002; Matsui and Maeda 2018). Females deposit their eggs in lentic environments such as ponds (Govindarajulu et al. 2006), and the larvae feed on small aquatic invertebrates and algae (Ruibal and Laufer 2012). Following metamorphosis, juveniles and adults inhabit areas in and around ponds, preying on animals smaller than themselves (Flynn et al. 2017; Oda et al. 2019). Gut and stomach content analyses of juveniles and adults frequently reveal predation on both aquatic and terrestrial native species (Silva et al. 2009; Barrasso et al. 2009; Sarashina 2016; Flynn et al. 2017; Oda et al. 2019; Matsumoto et al. 2020). Some studies have reported that the introduction of *A*. *catesbeiana* has led to declines in native animals through direct predation (Kats and Ferrer 2003; Li et al. 2011). Juvenile *A*. *catesbeiana* were collected between July and September 2024 from the pond in the Kinki region where adult *R. attenuata* were commonly observed (Figure 1A,B). Frogs were kept in plastic containers (length: 120 mm; width: 85–190 mm; height: 130 mm) at 25°C. They were fed live mealworms [i.e., larvae of *Tenebrio molitor* Linnaeus (Coleoptera: Tenebrionidae)] and nymphs and adults of the cockroach *Periplaneta lateralis* Walker (Blattodea: Blattidae) as prey (Sugiura and Date 2022; Sugiura and Hayashi 2024, 2025a).

The native frog species *P. nigromaculatus* , *Pelophylax porosus* (Cope), *Glandirana rugosa* (Temminck and Schlegel) (Anura: Ranidae), *Fejervarya kawamurai* Tjong, Matsui, Kuramoto, Nishioka, and Sumida (Anura: Dicroglossidae), and *Dryophytes japonicus* (Günther) (Anura: Hylidae) occur in ponds and rice paddies in Japan (Matsui and Maeda 2018). These frogs prey on smaller aquatic and terrestrial invertebrates (Hirai and Matsui 1999, 2000a, 2000b, 2001a, 2001b; Sarashina et al. 2011; Sano and Shinohara 2012; Sarashina 2016; Matsui and Maeda 2018; Baba et al. 2023) and are frequently found in the same habitats as adult *R. attenuata* (Figure 1C,D; Sugiura 2020b). Juveniles and adults of these species were collected from the Kinki, Chubu, and Kanto regions of Japan between May 2019 and September 2024, as follows: *P. nigromaculatus* from three sites (Kinki); *P. porosus* from one site each in Kanto and Chubu; *G. rugosa* from two sites (Kinki) and one site (Chubu); *F. kawamurai* from three sites (Kinki), one site (Chubu), and one site (Kanto); and *D. japonicus* from six sites (Kinki). Frogs were housed in plastic containers (length: 120–140 mm; width: 80–190 mm; height: 100–130 mm) at 25°C and were fed live mealworms and cockroaches (Sugiura 2020b; Sugiura and Hayashi 2024).

The snout–vent length and body weight of each frog were measured to the nearest 0.01 mm and 0.1 mg, respectively, using electronic slide callipers and the electronic balance. Each frog was used only once in the following observations.

### 2.2 Behavioural Observations

I conducted intermittent observations of the defensive behaviour of adult *R. attenuata* against six frog species under laboratory conditions (25°C) between September 2019 and March 2025. The procedures largely followed those described in Sugiura (2020b). To ensure a standardised hunger level, frogs that had not eaten prey for more than 24 h were used (Honma et al. 2006; Sugiura and Hayashi 2023, 2024, 2025b).

Each frog was placed in a transparent plastic container (length: 120 mm; width: 85–190 mm; height: 130 mm). An adult *R. attenuata* was then introduced into the frog's field of view. The behaviours of the frog and beetle were recorded using digital cameras (iPhone XS or iPhone 12 Pro Max, Apple Inc., Cupertino, CA, USA) and digital video cameras (Handycam HDR‐PJ790V or FDR‐AX45, Sony Corp., Tokyo, Japan). If the frog did not respond to the beetle, I moved the beetle using forceps or a stick. If the frog did not attack the beetle, a palatable prey item (a mealworm) was offered after the rejection. Frogs that consumed the mealworm after rejecting the beetle were considered to have ignored *R. attenuata* . Data from frogs that did not consume the mealworm were excluded from analysis. When a frog appeared to have swallowed a beetle, I opened its mouth with forceps to confirm the ingestion. Frogs that had swallowed beetles were then transferred to transparent plastic containers (length: 55–120 mm; width: 35–190 mm; height: 45–130 mm). The emergence of the beetle from the frog, or its excretion, was recorded using digital video cameras (Handycam HDR‐PJ790V, HDR‐CX630V, FDR‐AX45, or FDR‐AX60, Sony Corp.). When a beetle emerged from the frog, its condition (dead or alive) was recorded. The total time required for passage through the digestive tract (i.e., transit time), from ingestion to emergence (or excretion), was determined to the nearest 1 min based on the video recordings. A total of 143 beetle–frog interactions were observed, involving 143 *R. attenuata* (body length: 3.5–5.2 mm; body weight: 4.5–11.3 mg) and the following frog species: 24 *A*. *catesbeiana* (snout–vent length: 37.1–73.8 mm; body weight: 4446.5–39583.3 mg), 21 *P. nigromaculatus* (22.5–74.2 mm; 849.8–36621.7 mg), 22 *P. porosus* (25.1–44.3 mm; 1178.2–7555.3 mg), 22 *G. rugosa* (22.3–44.9 mm; 814.7–8849.3 mg), 32 *F. kawamurai* (20.3–44.3 mm; 570.7–5872.8 mg), and 22 *D. japonicus* (21.5–39.7 mm; 765.9–6072.5 mg). Of these observations, 30% (43 *R. attenuata* , 15 *P. nigromaculatus* , 3 *P. porosus* , 4 *G. rugosa* , 11 *F. kawamurai* , and 10 *D. japonicus* ) were previously presented in Sugiura (2020c) and were reused in this study.

All observations were conducted in accordance with the Kobe University Animal Experimentation Regulations (Kobe University's Animal Care and Use Committee, Nos. 30–01, 2023–03). The frogs were not harmed during the experiments. All transportation and laboratory maintenance involving the American bullfrog *A*. *catesbeiana* were conducted with permission from the Kinki Regional Environmental Office of the Ministry of the Environment, Government of Japan (Permit No. 20000085), as this species is officially designated as an invasive non‐native species under Japan's Invasive Alien Species Act, which regulates the transport and keeping of live individuals.

### 2.3 Data Analysis

Generalised linear models (GLMs) with a binomial distribution and a logit link function were used to investigate which factors influenced the escape success of adult *R. attenuata* from the frog cloaca. The response variable was whether an adult beetle successfully escaped from the frog's cloaca (1) or not (0). Explanatory variables included *R. attenuata* size (body length or weight), frog species, and frog size (snout–vent length or body weight).

GLMs with a Poisson distribution and a log link were also used to investigate which factors influenced the time required for *R. attenuata* to pass through the frog digestive tract (i.e., total transit time). The response variable was total transit time (min). Explanatory variables included the condition of the beetle upon emergence (alive or killed), beetle body size (length or weight), frog species, and frog size (snout–vent length or weight).

Because both beetle body length and body weight, as well as frog snout–vent length and body weight, were positively correlated, the two size metrics were not included simultaneously in the same model in either analysis. Instead, separate models were constructed with either body length or body weight for beetles, and either snout–vent length or body weight for frogs, to avoid collinearity.

Data from frogs that did not swallow beetles were excluded from these analyses. All body size measurements were log‐transformed prior to analysis. For the binomial GLMs, residual deviance was similar to the residual degrees of freedom, indicating that overdispersion was not present. For the Poisson GLMs, however, overdispersion was detected; therefore, a negative binomial distribution was used in place of a Poisson distribution when the residual deviance was substantially larger than the residual degrees of freedom. All statistical analyses were conducted using R version 4.3.1. GLMs with a negative binomial distribution were implemented using the MASS package version 7.3–60. For all GLM analyses, odds ratios (ORs) or incidence rate ratios (IRRs) and their 95% confidence intervals (CIs) were calculated by exponentiating the model coefficients and their standard errors. A significance level of 0.05 was used for all tests.

## 3 Results

Most frogs attacked adult *R. attenuata* , although a few individuals ignored them (Table 1). Five frog species (87.5–100.0%) successfully swallowed adult beetles, whereas 28.1% of *F. kawamurai* individuals spat them out immediately after taking them into their mouths (Table 1).

**TABLE 1:** Behavioural responses of frogs to *Regimbartia attenuata*.

Frog species	Frog behaviour *n*						
	Ignore	Spit out^a^	Regurgitate^b^	Swallow *n* (%)			Total
				Digest	Escaped from vent^c^	Subtotal	
*Aquarana catesbeiana*	3	0	0	6 (28.6)	15 (71.4)	21 (100.0)	24
*Pelophylax nigromaculatus*	0	0	0	2 (9.5)	19 (90.5)	21 (100.0)	21
*Pelophylax porosus*	0	1	0	5 (23.8)	16 (76.2)	21 (100.0)	22
*Glandirana rugosa*	0	1	0	9 (42.9)	12 (57.1)	21 (100.0)	22
*Fejervarya kawamurai*	2	9	0	3 (14.3)	18 (85.7)	21 (100.0)	32
*Dryophytes japonicus*	0	0	1	2 (9.5)	19 (90.5)	21 (100.0)	22

^a^ ‘Spit out’ indicates that frogs spat out adult *R*. *attenuata* before swallowing them.

^b^ ‘Regurgitate’ indicates that frogs vomited adult *R*. *attenuata* after swallowing them.

^c^ ‘Escaped from vent’ indicates that adult *R*. *attenuata* escaped alive from the frog's vent after ingestion.

**FIGURE 3:**
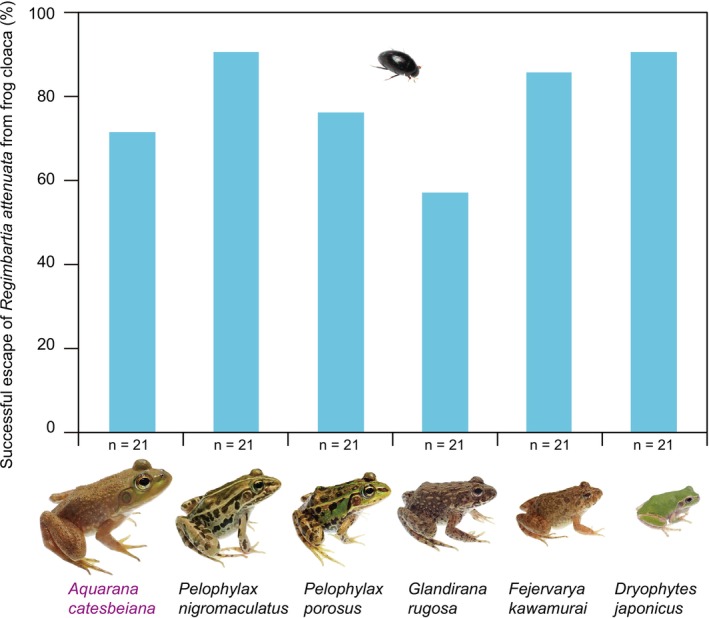
Escape success rates of adult *Regimbartia attenuata* from the cloaca of six frog species. The American bullfrog (*Aquarana catesbeiana*, shown in purple) is a non‐native species in Japan, while the other five frog species are native. Photo credits: Shinji Sugiura.

Of the adult *R. attenuata* swallowed by the non‐native bullfrog *A*. *catesbeiana*, 71.4% escaped alive through the digestive tract and exited from the cloaca (Table 1; Figure 3; Video 1). Among the beetles swallowed by native frog species, 57.1%–90.5% escaped alive through the cloaca (Table 1; Figure 3; Video 2). The success rate of *R. attenuata* escaping from *A*. *catesbeiana* (71.4%) was not significantly different from the escape success rates from native frog species (57.1%–90.5%; Table 2; Figure 3). In addition, neither *R. attenuata* size nor frog size significantly influenced the escape success rate (Table 2).

**VIDEO 1:** Escape of an adult *Regimbartia attenuata* from the cloaca of the non‐native bullfrog *Aquarana catesbeiana*. The beetle (body length: 5.0 mm) was excreted alive, along with a faecal pellet from the cloaca of the juvenile *A*. *catesbeiana* (snout–vent length: 48.2 mm) 72 min after ingestion. This video shows a representative individual trial. Video credit: Shinji Sugiura. Video content can be viewed at https://onlinelibrary.wiley.com/doi/10.1002/ece3.72477.

**VIDEO 2:** Escape of an adult *Regimbartia attenuata* from the cloaca of the native frog *Pelophylax porosus* . The beetle (body length: 4.0 mm) emerged from the cloaca of the juvenile *P. porosus* (snout–vent length: 40.5 mm) 166 min after ingestion. This video shows a representative individual trial. Video credit: Shinji Sugiura. Video content can be viewed at https://onlinelibrary.wiley.com/doi/10.1002/ece3.72477.

**TABLE 2:** Results of GLM testing effects of beetle size, frog species and frog size on escape success of *Regimbartia attenuata*.

Response variable	Explanatory variable	Estimate	SE	z	OR	95% CI for OR	p
(A) Effects of beetle body length, frog snout–vent length and frog species							
Escape success^a^	Intercept	6.06	7.00	0.87	429.80	0.00–621328700	0.386
	Beetle body length	−1.26	4.01	−0.31	0.28	0.00–680.50	0.753
	Frog snout–vent length	−0.84	1.30	−0.65	0.43	0.03–5.64	0.517
	Frog species (*P*. *nigromaculatus*)^b^	1.18	0.91	1.30	3.27	0.62–25.36	0.193
	Frog species (*P*. *porosus*)^b^	−0.13	0.85	−0.15	0.88	0.16–4.75	0.878
	Frog species (*G*. *rugosa*)^b^	−0.95	0.78	−1.22	0.39	0.08–1.76	0.224
	Frog species (*F*. *kawamurai*)^b^	0.53	0.92	0.58	1.70	0.29–11.42	0.562
	Frog species (*D*. *japonicus*)^b^	0.93	1.06	0.87	2.53	0.34–24.86	0.383
(B) Effects of beetle weight, frog weight and frog species							
Escape success^a^	Intercept	1.48	4.58	0.32	4.39	0.00–47317.75	0.747
	Beetle weight	1.69	1.55	1.10	5.44	0.25–114.43	0.273
	Frog weight	−0.46	0.39	−1.18	0.63	0.29–1.35	0.236
	Frog species (*P*. *nigromaculatus*)^b^	1.29	0.92	1.41	3.65	0.67–28.88	0.159
	Frog species (*P*. *porosus*)^b^	−0.06	0.83	−0.08	0.94	0.18–4.91	0.936
	Frog species (*G*. *rugosa*)^b^	−0.94	0.78	−1.21	0.39	0.08–1.76	0.225
	Frog species (*F*. *kawamurai*)^b^	0.42	0.93	0.45	1.52	0.25–10.29	0.651
	Frog species (*D*. *japonicus*)^b^	0.84	1.06	0.79	2.31	0.31–22.57	0.430

^a^ A binomial error distribution was used.

^b^ The non‐native frog *Aquarana catesbeiana* was used as the reference category.

Among the 91 live *R. attenuata* individuals clearly recorded escaping from the frog's cloaca, 89 (97.8%) exited head first (Videos 1 and 2), while the remaining two (2.2%) exited posterior end first. Some live beetles were frequently excreted together with faecal pellets (Video 1), whereas others were expelled alone, without any accompanying faecal material (Video 2).

**FIGURE 4:**
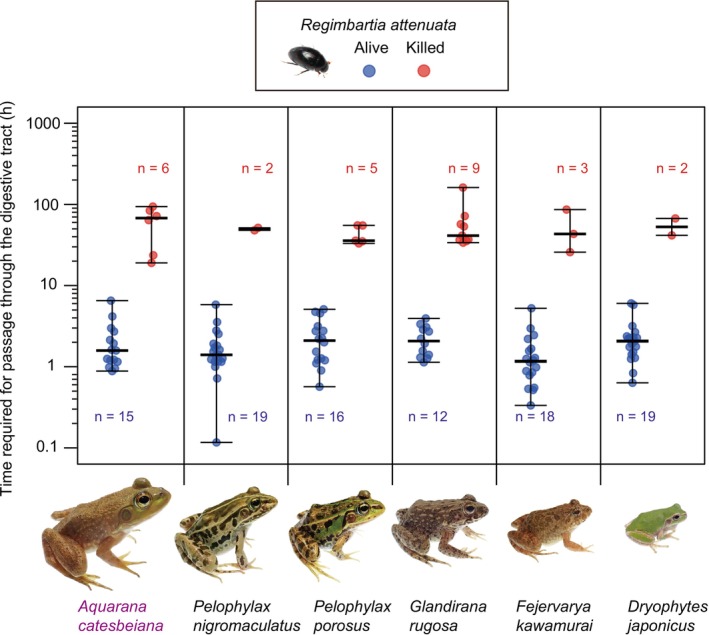
Time required for adult *Regimbartia attenuata* to pass through the digestive tract of six frog species. The American bullfrog (*Aquarana catesbeiana*, shown in purple) is a non‐native species in Japan, whereas the other five frog species are native. Each point represents an individual *R. attenuata* excreted from a frog's cloaca: Blue circles indicate beetles that survived passage (alive), and red circles indicate beetles killed during digestion. The vertical axis is on a logarithmic scale (log_10_). Horizontal lines represent the minimum, median (thick line), and maximum values for each frog species. Photo credits: Shinji Sugiura.

Live *R. attenuata* escaped from the frog cloaca 7–391 min (0.1–6.5 h) after ingestion (Figure 4). In contrast, undigested body parts of beetles that died inside the frogs were excreted 19.0–161.2 h after ingestion (Figure 4). The total transit time through the digestive tract was much shorter for live beetles than for those that died inside frogs (Table 3). While frog species and size did not significantly influence transit time, *R. attenuata* weight had a significant effect (Table 3); specifically, transit time increased with increasing beetle weight (Table 3B).

**TABLE 3:** Results of GLM testing effects of beetle survival, beetle size, frog species and frog size on transit time of *Regimbartia attenuata*.

Response variable	Explanatory variable	Estimate	SE	*z*	IRR	95% CI for IRR	*p*
(A) Effects of beetle survival, beetle body length, frog species and frog snout–vent length.							
Total transit time^a^	Intercept	4.96	1.49	3.32	141.99	7.61–2650.81	< 0.001
	Beetle survival (killed)^b^	3.27	0.13	25.86	26.35	20.57–33.77	< 0.001
	Beetle body length	0.45	0.85	0.53	1.57	0.30–8.27	0.594
	Frog snout–vent length	−0.20	0.28	−0.73	0.82	0.47–1.41	0.465
	Frog species (*P*. *nigromaculatus*)^c^	−0.20	0.18	−1.12	0.82	0.57–1.17	0.264
	Frog species (*P*. *porosus*)^c^	−0.03	0.20	−0.17	0.97	0.65–1.43	0.862
	Frog species (*G*. *rugosa*)^c^	−0.01	0.20	−0.05	0.99	0.67–1.46	0.960
	Frog species (*F. kawamurai*)^c^	−0.40	0.20	−1.98	0.67	0.45–1.00	0.048
	Frog species (*D*. *japonicus*)^c^	−0.03	0.21	−0.15	0.97	0.64–1.47	0.877
(B) Effects of beetle survival, beetle weight, frog species and frog weight							
Total transit time^a^	Intercept	3.68	0.97	3.80	39.81	5.96–265.93	< 0.001
	Beetle survival (killed)^b^	3.32	0.12	26.69	27.64	21.66–35.27	< 0.001
	Beetle weight	0.85	0.33	2.55	2.34	1.22–4.50	0.011
	Frog weight	−0.08	0.08	−0.92	0.93	0.79–1.09	0.359
	Frog species (*P*. *nigromaculatus*)^c^	−0.13	0.18	−0.71	0.88	0.62–1.25	0.480
	Frog species (*P*. *porosus*)^c^	0.05	0.19	0.26	1.05	0.72–1.53	0.794
	Frog species (*G*. *rugosa*)^c^	0.02	0.19	0.12	1.02	0.71–1.49	0.902
	Frog species (*F. kawamurai*)^c^	−0.37	0.20	−1.86	0.69	0.47–1.02	0.064
	Frog species (*D*. *japonicus*)^c^	0.03	0.21	0.17	1.04	0.69–1.55	0.868

^a^ Total transit time refers to the time required from frog ingestion to beetle escape (or frog defecation). A negative binomial error distribution was used instead of a Poisson distribution because the residual deviance was substantially larger than the residual degrees of freedom.

^b^ Live beetles were used as the reference category.

^c^ The non‐native frog *Aquarana catesbeiana* was used as the reference category.

## 4 Discussion

The prey behaviour of escaping through a predator's digestive tract and exiting via the cloaca has generally been considered a passive process, primarily dependent on the predator's defecation (Fair 1969; Barrentine 1988; Norton 1988; Brown 2007; Wada et al. 2012). In *R. attenuata* , adults that died within the frog digestive tract were typically egested during defecation, whereas live adults escaped through the cloaca much earlier than the usual defecation timing (Figure 4). Most adult *R. attenuata* exited head first from the frog cloaca (Videos 1 and 2). These results suggest that adult *R. attenuata* may actively move forward through the frog's digestive tract to facilitate escape.

In predators such as frogs that swallow prey whole, digestive fluids play a critical role in killing prey (Sugiura and Sato 2018). Therefore, rapid escape from the digestive tract is essential for prey survival (Sugiura and Sato 2018). Factors influencing escape success may include the activity level of adult *R. attenuata* , the distance that must be travelled through the digestive tract (i.e., the total length of the frog's digestive system), and the frog's digestive and killing capacity. In this study, I focused on the body size of both prey and predator as potential indicators of these factors. My results did not provide strong evidence that body size—of either *R. attenuata* or the frog—significantly affects the escape success rate (Table 2). These findings suggest that adult *R. attenuata* possess a high and consistent ability to escape via the frog cloaca, regardless of frog species or size.

### 4.1 Factors Influencing the Escape Success in *Regimbartia attenuata*

Unlike adults of beetle families Gyrinidae and Dytiscidae, adult Hydrophilidae (including *R. attenuata* ) lack defensive glands that secrete chemicals to deter predators (Dettner 2019). Furthermore, Sugiura (2020b) experimentally immobilised the mid‐ and hindlegs of adult *R. attenuata* and presented them to frogs; none of these beetles successfully escaped. These results suggest that *R. attenuata* adults rely primarily on active movement using their legs, rather than chemical defences, to escape from frog digestive systems.

To exit through the cloaca, adult *R. attenuata* must cause the frog to open its cloaca (Sugiura 2020b), which is normally kept closed by the sphincter muscle pressure (Waring et al. 1941). Some live *R. attenuata* were frequently excreted together with faecal pellets (Video 1), suggesting that the beetles stimulate the frog's hindgut and thereby induce defecation (Sugiura 2020b). Others were expelled alone, without any accompanying faecal material (Video 2). Moreover, live beetles exited much earlier than the expected timing of defecation (Figure 4). These findings are consistent with the possibility that adult *R. attenuata* stimulate the frog to open the cloaca, thereby inducing their own expulsion. To better understand how such behaviour contributes to high survival rates, future studies could directly observe *R. attenuata* movements within the frog's digestive tract using techniques such as high‐resolution X‐ray or CT imaging.

Like other adult aquatic beetles (Yee and Kehl 2015), adult *R. attenuata* can respire via a small air store beneath the elytra, where the spiracles open. This air store is connected to a ventral air film that functions as a compressible gill supported by water‐repellent hairs. Such a respiratory system could contribute to survival under the low‐oxygen conditions within the frog digestive tract, although this has not been directly tested. In addition, the hard exoskeleton of adult *R. attenuata* may offer protection against the frog's acidic or alkaline digestive fluids.

Furthermore, their aquatic behaviour—including rapid swimming, pushing through aquatic vegetation, and burrowing into or emerging from mud—may facilitate active movement through the digestive tract towards the cloaca, ultimately enabling escape through the cloaca.

The body size of adult *R. attenuata* ranged from 3.5 to 5.2 mm. The time required for passage through the frog digestive tract did not change with beetle body length (Table 3A), but increased with increasing beetle weight (Table 3B), suggesting that heavier beetles may escape later from the lethal digestive environment than lighter ones. However, body size (both length and weight) did not significantly influence escape success (Table 2). If the individual's body size is too small, the amount of air that can be stored beneath the elytra may be limited, which could reduce survival under anaerobic conditions in the digestive tract. Conversely, if the body size is too large, movement through the digestive tract or cloaca may be hindered. Therefore, the structure of the frog digestive tract may impose a selective pressure that constrains body size evolution in adult *R. attenuata* , though further testing is required to confirm this.

### 4.2 Effects of Predator Species and Size

Previous studies have shown that native insects defend themselves less successfully against the non‐native bullfrog *A*. *catesbeiana* than against native frogs (Sugiura and Hayashi 2024, 2025a; but see Sugiura and Date 2022). In the present study, I observed the successful escape of adult *R. attenuata* from at least six frog species, representing three families, including *A*. *catesbeiana*.

Notably, adult *R. attenuata* escaped from *A*. *catesbeiana* at a high frequency comparable to that observed for native frog species (Figure 3). Because *R. attenuata* and its congeners do not occur in North America, where *A*. *catesbeiana* originates (Hansen 1991; Mai et al. 2022), their interactions have not been shaped by coevolution over an evolutionary timescale. Therefore, rather than having evolved a defensive behaviour specifically against *A*. *catesbeiana*, it is more likely that the escape strategy developed through interactions with native frogs is also effective against this non‐native predator. This may be due to structural similarities in digestive tracts among frog species, regardless of taxonomic differences.

However, the wide confidence intervals of the odds ratios indicate a high degree of uncertainty in the estimates (Table 2). In particular, the comparison between the non‐native *A*. *catesbeiana* and native frog species may have been underpowered due to the limited sample sizes. Therefore, while my analyses did not detect significant interspecific differences, further studies with larger sample sizes will be necessary to draw more robust conclusions.

A number of *R. attenuata* carcasses were found in the faeces of wild‐caught juvenile *A*. *catesbeiana* (Figure 2). Typically, the presence of native species in the stomach contents of *A*. *catesbeiana* has been interpreted as evidence of its negative impact on native fauna (Hirai 2005; Wu et al. 2005; Hirai and Inatani 2008; Silva et al. 2009; Barrasso et al. 2009; Oda et al. 2019; Nakamura and Tominaga 2021). However, the presence of native prey in stomach contents does not necessarily indicate frequent predation success. High local densities of a given native species may increase encounters and ingestion rates, even if actual predation success is low (Sugiura and Date 2022). In fact, *R. attenuata* maintained high population densities even in the pond where *A*. *catesbeiana* was abundant (Figure 1A,B). Thus, the impact of non‐native bullfrogs on *R. attenuata* may be smaller than that on other aquatic insects, such as Gyrinidae (Sugiura and Hayashi 2024).

The present study did not find evidence that frog size significantly affects the escape success of adult *R. attenuata* (Table 2). Adult *R. attenuata* escaped from native frog species spanning a wide range of body sizes (snout–vent length: 22.1–64.3 mm), covering most of the size variation they are likely to encounter in Japan (Matsui and Maeda 2018). However, only juveniles of *A*. *catesbeiana* (snout–vent length: 37.1–73.8 mm) were used in this study, and the effects of larger adult bullfrogs were not examined. Adult *A*. *catesbeiana* can exceed 150 mm in snout–vent length (Nakamura and Tominaga 2021). In this study, *R. attenuata* failed to escape from frogs with snout–vent lengths greater than 70 mm, suggesting that escape from adult *A*. *catesbeiana* or other large frog species may be unlikely. Nevertheless, such large frogs are unlikely to actively prey on *R. attenuata* in the field.

## 5 Conclusions

This study demonstrated that adult *R. attenuata* can escape through the cloaca after surviving passage through the digestive tract of various frog species with different body sizes. It should be noted that adults of the five native frog species were included in this study, whereas only juvenile *A*. *catesbeiana* were examined. Therefore, my findings should be interpreted with caution when considering their applicability to adult *A*. *catesbeiana*.

The high escape success rate suggests that this behaviour represents a specialised defensive strategy adapted to evade frog predation. In contrast, frogs appear to have evolved few, if any, counter‐adaptations to prevent the escape of *R. attenuata* . The small, streamlined body shape of adult *R. attenuata* likely facilitates smooth passage through the digestive tract, minimising potential damage to the predator's internal organs. Therefore, in prey species capable of escaping through the predator's digestive system, counter‐defensive adaptations in predators may be less likely to evolve if the prey is small, of low nutritional value, and does not harm the digestive tract. In addition, potential constraints such as the frog's gut morphology, cloacal musculature, or digestive enzyme profiles may partly explain why counter‐adaptations have not evolved in frogs.

## Author Contributions

**Shinji Sugiura:** conceptualization (lead), data curation (lead), formal analysis (lead), funding acquisition (lead), investigation (lead), methodology (lead), project administration (lead), validation (lead), visualization (lead), writing – original draft (lead), writing – review and editing (lead).

## Conflicts of Interest

The author declares no conflicts of interest.

## Data Availability

The raw data are available at figshare: Sugiura S (Sugiura 2025b) Data from: Effects of predator species and size on prey escape success through the digestive tract. https://doi.org/10.6084/m9.figshare.28877027.
